# The role of ethical climate on teachers’ wellbeing: relationship with psychophysical wellbeing and general health

**DOI:** 10.3389/fpubh.2025.1631347

**Published:** 2025-10-16

**Authors:** Ivan Borrelli, Giacomo Angelini, Paolo Emilio Santoro, Maria Francesca Rossi, Maria Rosaria Gualano, Umberto Moscato, Caterina Fiorilli

**Affiliations:** ^1^Section of Occupational Health, Department of Health Surveillance and Bioethics, Catholic University of the Sacred Heart, Rome, Italy; ^2^Department of Human Sciences, University of LUMSA, Rome, Italy; ^3^Catholic University of the Sacred Heart, Rome, Italy; ^4^Saint Camillus International University of Health Sciences, UniCamillus, Rome, Italy

**Keywords:** ethical climate, anxiety, depression, general health, teachers

## Abstract

**Introduction:**

Previous studies have highlighted that the dissonance between workplace and personal ethics can lead to poorer mental health for employees. However, studies addressing the relationship between teachers’ wellbeing and workplace ethics are scarce. This study aims to investigate the relationship between ethical climate and mental and physical health outcomes in teachers.

**Methods:**

A cross-sectional study was conducted with 255 Italian teachers (88.63% female; mean age = 47.09 years). The study assessed the relationships among workplace ethics, general and mental health, and daytime sleepiness by descriptive analysis and multiple logistic regression models.

**Results:**

The study involved 255 Italian teachers recruited from schools of various grades (Female = 88.63%; Mean age = 47.09; SD = ±10.55). Most participants showcased “Good” (50.59%) or “Excellent” (42.75%) workplace ethics; 30.79% of participants reported anxiety, 27.06% depression, and 24.31% stress. Only 7.84% of participants scored over the cut-off for the general health questionnaire, and 26.67% reported daytime sleepiness. Using the logistic regression models, a significant correlation for workplace ethics was detected with the DASS-21 stress (*p* = 0.002) and depression (*p* = 0.011) subscales.

**Conclusion:**

The ethical climate in educational settings influences teachers’ mental health, particularly by reducing stress and depression. Enhancing the ethical environment in educational organizations could improve teacher wellbeing.

## 1 Introduction

Workplace ethical climate (WEC) refers to the environment that fosters moral reasoning, mutual respect, and trust within an organization. It represents a shared understanding of morally appropriate employee behavior (1). Ethical climate encompasses the rules and principles that guide individual and group behavior in a workplace setting (2). WEC provides a nuanced view of how work environments are shaped by specific ethical values (like benevolence and principles) and decision-making levels (individual to universal), influencing collective ethical behaviors within work environments to foster trust, commitment, and a positive climate (3). Previous findings in helping professional settings significantly support the hypothesis that the ethical dissonance between organizational and personal ethical values negatively impacts employee mental health (4, 5). For example, lower ethical standards in the workplace are associated with increased work-related stress, depression, anxiety, and burnout (6–9). Employees with higher moral standards tend to experience significantly lower stress levels over time, highlighting the importance of maintaining a supportive ethical climate (10). The current study addresses this association within educational institutions, which is rarely investigated.

### 1.1 A new model to evaluate ethical climate at school

Recently, the Corporate Ethical Virtues (CEV) model, developed by Kaptein (11), emphasized that ethical behavior is fostered by cultivating specific moral qualities within organizations. Influenced by Victor and Cullen’s ethical climate theory and Hofstede’s cultural dimensions framework, Kaptein designed the CEV model as a multidimensional approach to ethical culture. More in detail, Victor and Cullen’s model introduced the concept that an organization’s ethical climate, formed through shared perceptions of ethical behavior, impacts decision-making and ethical outcomes in business settings (12). In the same way, Hofstede’s research explored how organizational and national cultures shape individual values, providing a basis for understanding cultural influences on organizational ethics (13). Kaptein extended these foundational ideas, proposing that an ethical workplace is achieved through specific organizational virtues that enable and reinforce ethical behavior. These virtues are interdependent qualities that create an environment where ethical actions align with organizational goals, promote trust, and deter misconduct. For example, organizations committed to ethical transparency define clear behavioral expectations and actively support and reward ethical behavior in their employees.

The CEV model has been empirically validated and recently adapted in a shortened Italian version for assessing and fostering an ethical culture in diverse contexts through a self-report questionnaire (14). Investigating the CEV teachers’ perception of their school context could promote attention toward ethical behaviors and virtues. Due to the nature of the educational settings, the impact of ethical climate on personal and organizational decision-making processes is expected to play a crucial role. The school environment is characterized by shared ethical values that can influence the entire organization (15). In general workplace contexts, it has been highlighted that job performance is related to organizational climate, underlining how interactions at the company level could affect efficiency and performance (16). Hence, for teachers, a shared set of ethical values may help resolve conflicts constructively, manage disputes, and foster understanding and reconciliation rather than division and hostility. Additionally, ethical values may build teachers’ trust, improve communication and collaboration, and create a safe and nurturing educational environment. Indeed, a positive school climate can enhance organizational performance, teaching quality, and student outcomes (17). While the validation of the CEV questionnaire was performed on office worker, a questionnaire based on the same model was used to study ethical climate in university teachers (18). Furthermore, the dimensions of ethical climate in (non-university) schools were investigated across multiple countries by Shapira-Lishchinsky, highlighting that school ethical climate largely overlapped with the CEV model (19). In this study, the author underlines that studying ethical climate in teachers by applying this model could not only be appropriate, but overall beneficial, in order to better understand what an ethical climate in school would entail, and to promote ethical behavior in teachers (which would in turn benefit the students as well as the overall school’s ethical climate) (19).

Ethical climate in schools has been investigated in students as well. University students reportedly have a similar corporate ethical climate’s perception to their teachers (20), and the overall school’s ethical climate influences the unethical behavior of university students, reducing it (21). Furthermore, in primary schools’ ethical climate affects the trust that teachers and students have in each other, as well as motivation in teachers (22). The effect of teacher’s ethical climate may therefore affect students as well, and improving it could benefit them as well.

In general, positive mental health outcomes in teachers have been showcased to improve their relationship with students, their engagement, and students’ performance. A recent literature review has showcased that positive health outcomes in teachers improve their engagement and resilience, correlating to longer stays in their roles; furthermore, positive mental health in teachers correlates to stronger relationships with their students and improve students’ outcomes (23). However, it has been highlighted in a literature review by Ozturk et al., that most studies currently available on teachers’ wellbeing do not consider a holistic approach to their mental health (encompassing personal and professional aspects, positive and negative outcomes), but this appears instrumental in evaluating the benefits that engaged and healthy teachers could have on the scholastic system (24). Evaluating workplace ethical climate in this context could offer a perspective on how to improve the wellbeing of teachers to foster a healthier and comprehensive approach to their mental health.

Previous studies have shown that teachers are more likely to adopt maladaptive behaviors when they perceive a misalignment between their personal and organizational ethics. Among these, the absenteeism rate is the most demonstrated (25). Moreover, a clash between individual and organizational ethical experiences can also undermine the intrinsic motivational experiences of teachers and result in reduced morale and professional commitment. Teachers will likely develop negative attitudes towards their workplace if school leaders and colleagues do not meet their ethical expectations. Concerning the Italian education system, teachers often work in under-resourced educational systems, viewed as a societal undervaluation of the teaching profession (26). In such conditions and worse ethical climates, educators may feel their work and ethical standards are undervalued. Understanding WEC’s influence in this educational framework thus offers insight into how the interventions for teacher retention, wellbeing, and improvements in education may be suitably addressed. Despite the wide findings supporting the negative impact of the misalignment between the organization and personal expectation’s ethical values, most studies focused on negative outcomes like teachers’ attitudes, motivation to leave, and maladaptive behaviors in their workplace context. It would be interesting and useful to analyze whether and to what extent these dimensions are also associated with teachers’ wellbeing.

### 1.2 Teachers’ wellbeing, consequences, and antecedents in the workplace setting

Teachers’ wellbeing in the workplace directly impacts their psychological and physical health, substantially impacting educational environments when it declines. Teachers’ wellbeing refers to a state of physical, psychological, and emotional health that enables educators to perform effectively and engage meaningfully with their students and the school environment (27–28). A scoping review investigating teacher’s mental health has reported on the prevalence of mental health disorders in teachers: from 25.1 to 74.0% for burnout, from 8.3 to 87.1% for stress, from 38.0 to 41.2% for stress, from 4.0 to 77.0% for depression (29). Evidence shows that stressful working conditions, exacerbated by factors such as high workload and lack of support, are associated with elevated psychological distress in teachers, leading to anxiety, depression, and burnout (30–32). Such mental strain often results in reduced job satisfaction and compromised quality of work, which, over time, contributes to a higher incidence of presenteeism (i.e., teachers attending work despite poor mental health) and a decision to leave the profession [e.g., Gadermann et al. (33)]. A recent systematic review has also highlighted that burnout in teachers is consistently associated with various symptoms and illnesses, as well as with cortisol dysregulation and systemic inflammation (34).

Physical manifestations of this psychological toll are also evident. The prevalence of sleep disorders among teachers, including insomnia and daytime sleepiness, not only impacts their health but also diminishes their resilience in coping with daily occupational stress. These disturbances in sleep contribute to decreased concentration, irritability, and, ultimately, impaired teaching efficacy [e.g., Crain et al. (35) and Huyghebaert et al. (36)]. This degradation of teachers’ wellbeing does not merely affect individuals but radiates out, affecting student wellbeing and the overall school climate (37, 38). For instance, studies have shown that teachers’ mental health influences students’ wellbeing and academic achievement, indicating a significant trickle-down effect of teachers’ wellbeing on student outcomes (39), which is particularly important when considering that negative mental health outcomes in high school and university students are highly prevalent, as highlighted by recent studies (40, 41). Given these findings, the adverse consequences of low teacher wellbeing extend beyond individual health issues to influence broader educational effectiveness and student mental health (42, 43). This construct encompasses multiple dimensions, including job satisfaction, professional fulfillment, and a sense of personal accomplishment, all of which contribute to an educator’s ability to teach effectively and maintain resilience against occupational stressors (44), which is especially relevant when considering how resilience and personal resources can reduce the effects of high job demands (45).

In summary, existing literature has investigated how teachers’ workplace climate supports their wellbeing, including social resources and supportive environments. More specifically, a recent meta-analysis (42) emphasizes the role of a positive workplace climate, where social relationships and supportive leadership significantly contribute to better teacher wellbeing.

### 1.3 Gap in current knowledge

Despite the relevant effect of the WEC on educational settings, research addressing this topic is limited. Furthermore, most studies have focused on the relationship between WEC and job aspects such as job performance, task adherence, and job satisfaction. To the authors’ knowledge, only one previous study has focused on the relationship between teachers’ burnout and WEC perception [e.g., Doğanay (46)], leaving out aspects such as teachers’ physical and mental health symptoms. Preventing illness among teachers is essential for safeguarding their health, maintaining teaching quality, and managing public spending related to teacher stress and burnout (31, 47, 48).

While general school climate has been thoroughly studied, this study aims to add to the existing scientific literature concerning ethical climate in the context of teachers’ occupational health and its relationship with mental wellbeing. Particularly, with the aforementioned correlation with organizational ethics and workplace behaviors (engagement, absenteeism, etc.) in mind, this study aims to fill the gap in current knowledge regarding workplace ethics in teachers and its correlation to their mental health and general wellbeing.

### 1.4 Aims and hypotheses

Based on the theoretical framework outlined, the current study aims to fill this gap by exploring the role of the ethical climate within educational institutions on teachers’ mental and physical health outcomes. In detail, we investigated the role of the workplace ethical climate (WEC) in the school setting, focusing on its potential to mitigate or intensify teachers’ stress levels and overall wellbeing.

We hypothesize that:

*H1*: Teachers’ WEC is negatively correlated with their levels of depression, anxiety, and stress (H1a). Additionally, we expect a positive correlation between WEC and teachers’ overall wellbeing (H1b) and a negative correlation with sleep disturbances (H1c).

*H2*: Teachers’ levels of depression (H2a), anxiety (H2b), and stress (H2c), predict their WEC.

## 2 Materials and methods

### 2.1 Participants and procedure

The study involved 255 Italian teachers recruited from schools of various educational levels (middle and high school). The sample comprised 226 females (88.63%) and 29 males (11.37%), with a mean age of 47.09 years (SD = 10.55; range 22–65). The gender ratio and the age mean and range of the participants is representative of the Italian teaching population and consistent with the national average (49).

A cross-sectional study was conducted in line with other studies to evaluate psychological wellbeing in adults and workers (50, 51). The recruitment was conducted by recruiting various Italian middle and high schools; training and information courses were planned and carried out in these schools to inform teachers of the psychological and general risks associated with their professional field. During these courses, a questionnaire was administered to the teachers, who could freely choose to participate in the study. All participants were informed about the research objectives and provided voluntary informed consent for data collection and processing. No personal identifying information was collected, and the survey required all questions to be answered, resulting in no missing data. The study received ethical approval from the participating university’s Ethics Committee for Scientific Research (CERS) and was conducted under the principles of the Declaration of Helsinki.

### 2.2 Measures

#### 2.2.1 Corporate ethical virtues (CEV)

The ethical climate was assessed using the sCEVit questionnaire, an Italian adaptation of the Corporate Ethical Virtues (CEV) model by Victor and Cullen (12). The sCEVit consists of 24 items (e.g., “In my immediate working environment, I am sometimes asked to do things that conflict with my conscience”) rated on a 6-point Likert scale from 1 (“Not at all”) to 6 (“In detail”) (11, 14). The scale does not have a definitive cut-off point; thus, results were categorized into quartiles to indicate varying levels of perceived ethical climate: “Poor” (0–36), “Discrete” (37–72), “Good” (73–108), and “Excellent” (109–144). The choice to categorize the scores from this scale into quartiles was made to make the instrument easily usable in non-scientific settings, and thus in workplace settings, by administrators who may be less experienced in scientific research. A similar approach has been established for other risk models [i.e., HSE questionnaire (52)], diving scores into quartiles to identify those situations for which interventions are needed from situations where the score is still acceptable. In the current study, CEV has demonstrated satisfactory psychometric properties, with adequate internal consistency and excellent Cronbach’s *α* (α = 0.92).

#### 2.2.2 Depression anxiety and stress scale (DASS-21)

Psychological distress was measured using the Depression Anxiety and Stress Scale (DASS-21), validated in Italian by Bottesi et al. (53). This 21-item scale (e.g., “I found it hard to wind down”) assesses symptoms of depression, anxiety, and stress on a 4-point Likert scale from 0 (“Did not apply to me at all”) to 3 (“Applied to me very much or most of the time”). The cut-offs used were for the depression subscale, absence ≤ 9, presence ≥ 10; for the anxiety subscale, absence ≤ 7, presence ≥ 8; for the stress subscale, absence ≤ 14, presence ≥ 15 (26). In the current study, DASS-21 has demonstrated satisfactory psychometric properties, with adequate internal consistency and excellent Cronbach’s *α* (α = 0.95).

#### 2.2.3 General health questionnaire (GHQ-12)

Teachers’ general health was evaluated by the General Health Questionnaire (GHQ-12) validated in Italian by Piccinelli et al. (54) (e.g., “Are you able to concentrate?”), is a 12-item questionnaire which uses a 4-point Likert scale from 0 (“Always”) to 3 (“Never”), with scores indicating normal health (0–14) or the presence of distress (15 or above) (55). In the current study, DASS-21 has demonstrated satisfactory psychometric properties, with adequate internal consistency and good Cronbach’s *α* (α = 0.82).

#### 2.2.4 Epworth sleepiness scale (ESS)

Daytime sleepiness was evaluated using the Epworth Sleepiness Scale (ESS) (56), validated in Italian by Vignatelli et al. (57). This scale includes eight items (e.g., “How likely are you to doze off or fall asleep in the following situations?”) rated on a 4-point Likert scale from 0 (“No chance of dozing”) to 3 (“High chance of dozing”). The ESS is used to assess the likelihood of falling asleep in various situations during the day, with scores above 10 indicating excessive daytime sleepiness (57, 58). In the current study, DASS-21 has demonstrated satisfactory psychometric properties, with adequate internal consistency and acceptable Cronbach’s *α* (*α* = 0.68).

### 2.3 Analytic strategy

Descriptive statistics were used to summarize the socio-demographic characteristics of the participants and the distribution of scores across the various questionnaires. Cronbach’s alpha evaluated the internal consistency of the scales. Given the non-normal distribution of key variables, as indicated by Shapiro-Francia tests, we explicitly excluded parametric statistical tests that assume normality (e.g., *t*-tests or ANOVA) from our analyses. Instead, we used Pearson’s chi-square test and multinomial logistic regression models, which are statistically appropriate methods for non-normally distributed data. No data transformations were applied, as the categorical nature of both independent and dependent variables justified the analytical approach adopted. More in detail, Pearson’s chi-square tests were employed to assess the associations between the ethical climate and the dimensions of psychological distress (depression, anxiety, and stress), general health, and daytime sleepiness.

Multinomial logistic regression models were developed when the univariate correlation was statistically significant to explore the predictive relationships between WEC and psychological distress outcomes (significantly correlated). The models were adjusted for potential confounding variables, including gender, age, type of employment contract (permanent or fixed term), and the number of students in the classroom (59). Adjusted Relative Risks (RRs) and 95% confidence intervals (CIs) were calculated. Statistical significance was determined at a *p*-value of less than 0.05. Data were managed using Microsoft Excel, and statistical analyses were performed using Stata IC software, version 16.

## 3 Results

### 3.1 Preliminary analysis

The preliminary analysis revealed that the data from the administered questionnaires were not normally distributed. This was determined using S-Francia tests, which indicated significant deviations from normality for variables such as age, DASS-21 scores (depression, anxiety, and stress), sCEVit scores, and ESS scores.

### 3.2 Descriptive statistics

Table 1 summarizes the socio-demographic characteristics of 255 Italian teachers, predominantly female (88.63%), with an average age of 47.09 years (SD = 10.55). Education levels vary: 34.12% completed high school, 10.59% have a bachelor’s degree, 37.65% hold a degree, and 17.65% attained a master’s or Ph.D. Most participants (76.08%) are on permanent contracts, with 23.92% on fixed-term contracts, teaching across nursery (15.69%), elementary (55.29%), middle (16.86%), and high school (12.16%) levels.

**Table 1:** Socio-demographic characteristics of the included sample.

Variable	Mean	SD
Sample = 255		
Age	47.09	10.55

Variable	Freq.	Percent
Gender		
Male	29	11.37
Female	226	88.63
Educational level		
High School	87	34.12
Bachelor’s degree	27	10.59
Degree	96	37.65
Master/PhD	45	17.65
Contract		
Permanent	194	76.08
Fixed-term	61	23.92
School type		
Nursery	40	15.69
Elementary	141	55.29
Middle	43	16.86
High	31	12.16
Seniority		
<6	61	23.92
6–10	53	20.78
11–20	46	18.04
21–30	53	20.78
31	42	16.47
Region		
South	27	10.59
Center	39	15.29
North	189	74.12
Learning disorder		
No	34	13.33
Yes	221	86.67
Disabilities		
No	42	16.47
Yes	213	83.53

Experience distribution: 23.92% have <6 years, 20.78% have 6–10 years, 18.04% have 11–20 years, 20.78% have 21–30 years, and 16.47% have >30 years. Geographically, most are from Northern Italy (74.12%), with smaller groups from Central (15.29%) and Southern Italy (10.59%)—additionally, 86.67% work with students with learning disorders and 83.53% with students with disabilities.

The scores from the questionnaires administered are reported in Table 2.

**Table 2:** Scores distribution based on cut-offs.

sCEVit	Freq.	Percent
Poor	0	0
Discrete	17	6.67
Good	129	50.59
Excellent	109	42.75
DASS-21 Stress		
No	177	69.41
Yes	78	30.59
DASS-21 Depression		
No	186	72.94
Yes	69	27.06
DASS-21 Anxiety		
No	193	75.69
Yes	62	24.31
GHQ-12		
No	235	92.16
Yes	20	7.84
ESS		
No	187	73.33
Yes	68	26.67

### 3.3 Correlation analysis (research hypothesis H1)

Table 3 summarizes the correlations between ethical climate scores (sCEVit) and psychological distress dimensions (stress, anxiety, depression) from the DASS-21, categorized by ethical climate rating (“Discrete,” “Good,” “Excellent”) (hypothesis H1a). Higher ethical climate scores correlate with lower stress, with 32.56% reporting stress in the “Good” category compared to 22.94% in the “Excellent” category. No significant correlation was found with anxiety (*p* = 0.831), showing similar levels across categories. However, a significant negative correlation was observed with depression (*p* = 0.011), as 34.11% in the “Good” category reported depression versus 17.43% in the “Excellent” category. These findings suggest that a positive ethical climate is correlated with reduced stress and depression but does not significantly correlate with anxiety. Hypothesis H1 was accepted for stress and depression but rejected for anxiety.

**Table 3:** Correlation between ethical climate (sCEVit) and psychological distress (DASS-21) (*significative correlation with sCEVit).

sCEVit	Stress*			Anxiety			Depression*		
	No	Yes	Total	No	Yes	Total	No	Yes	Total
Poor	0	0	0	0	0	0	0	0	0
Discrete	6	11	17	12	5	17	11	6	17
Good	87	42	129	97	32	129	85	44	129
Excellent	84	25	109	84	25	109	90	19	109
Total	177	78	255	193	62	255	186	69	255

Pearson *χ*^2^ = 12.56; Prob = 0.002; Mean = 6.03; SD ± 4.5. Pearson *χ*^2^ = 0.37; Prob = 0.831; Mean = 2.61; SD ± 3.45. Pearson *χ*^2^ = 8.95; Prob = 0.011; Mean = 3.23; SD ± 3.95.

Table 4 examines the relationship between ethical climate scores (sCEVit) and two health outcomes: general health (GHQ-12) (hypothesis H1b) and daytime sleepiness (ESS) (hypothesis H1c). Concerning general health (GHQ-12), no significant correlation was found with ethical climate scores (*p* = 0.6788). Most participants reported normal health across both “Good” and “Excellent” ethical climate categories, with 93.58% indicating normal health and 6.42% reporting psychological distress in each category, suggesting that ethical climate variations are not significantly correlated with general health outcomes. Similarly, no significant correlation was observed for daytime sleepiness (ESS) (*p* = 0.6985). In the “Good” category, 24.81% reported excessive daytime sleepiness, compared to 29.36% in the “Excellent” category, with normal sleepiness levels reported by 75.19 and 70.64%, respectively. These results indicate that perceived ethical climate does not significantly correlate with daytime sleepiness among participants.

**Table 4:** Correlation between ethical climate (sCEVit) and general health (GHQ-12) and daytime sleepiness (ESS) (no significative correlation emerged).

sCEVit	GHQ			ESS		
	No	Yes	Total	No	Yes	Total
Poor	0	0	0	0	0	0
Discrete	1	16	17	13	4	17
Good	12	117	129	97	32	129
Excellent	7	102	109	77	32	109
Total	20	235	255	187	68	255

Pearson *χ*^2^ = 0.77 Prob = 0.6788; Mean = 20.27; SD ± 5.39. Pearson *χ*^2^ = 0.72 Prob = 0.6985; Mean = 5.11; SD ± 2.57.

Overall, a positive ethical climate does not significantly correlate with general health or daytime sleepiness in this sample of teachers.

### 3.4 Regression models (research hypothesis H2)

Table 5 shows multinomial regression results examining the relationship between ethical climate (sCEVit scores) and depression levels (DASS-21) (hypothesis H2a). This model was chosen due to a significant univariate correlation. As no participant scored “Poor” on the sCEVit, the “Good” category was set as the baseline, excluding the lower quartile. The table presents risk ratios (RR) and *p*-values for different ethical climate levels, adjusting for confounders like gender, age, contract type, and classroom size.

**Table 5:** Multinomial regression models for sCEVit scores and Depression subscale from DASS-21, pseudo *r*^2^ provides a global measure of model fit (significative correlation for crude and adjusted Depression scores to an “Excellent” score at the sCEVit).

sCEVit	Pseudo *r*^2^	*p*-values	DASS-21 subscale	RRR	St. Err.	*t*-value	*p*-value	[95% Confidence Interval]	Sig
Regression model									
Discrete	0.020	0.010	Depression^a^	0.052	0.54	0.10	0.923	−1.007 to 1.112	
	0.041	0.005	Depression^b^	1.035	0.57	0.06	0.95	0.351–3.048	
	0.052	0.003	Depression^c^	1.026	0.571	0.05	0.963	0.345–3.056	
	0.066	0.001	Depression^d^	1.001	0.561	0.00	0.998	0.334–3.003	
Good (base level)									
Excellent			Depression^a^	−0.897	0.313	−2.86	0.004	−1.511 to −0.283	***
			Depression^b^	0.415	0.131	−2.78	0.005	0.223–0.772	***
			Depression^c^	0.383	0.124	−2.97	0.003	0.204–0.722	***
			Depression^d^	0.395	0.128	−2.87	0.004	0.209–0.745	***

***p < 0.01, **p < 0.05, *p < 0.1.

a Crude.

b Adjusted for sex and age.

c Adjusted for sex, age, contract.

d Adjusted for sex, age, contract, student number.

The “Discrete” Ethical Climate category did not show a significant association with depression levels, as risk ratios were not statistically significant, suggesting that this level does not notably correlate with depression risk compared to the “Good” baseline.

For the “Excellent” Ethical Climate, results from Model A (Crude regression) indicate that participants with “Excellent” climate perceptions had a reduced risk of depression compared to the “Good” group (RR = 0.52, *p* = 0.004). This effect remained significant in adjusted models A, C, and D (RR = 0.41, *p* = 0.005) after controlling for various factors: Model B adjusted for gender and age, Model C for gender, age, and contract type, and Model D included gender, age, contract type, and classroom size. The consistency across models reinforces the robustness of this association.

**Figure 1:**
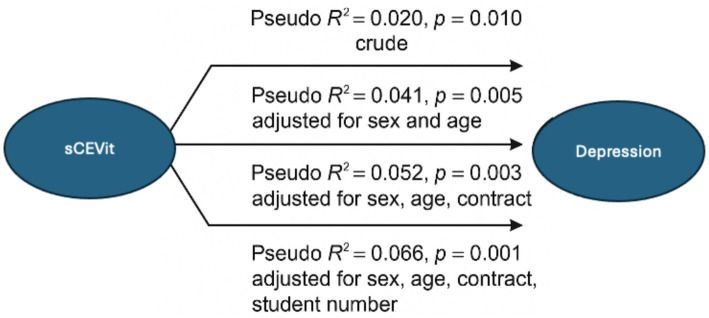
Conceptual diagram of the multinomial logistic regression model for Depression. The figure illustrates the association between sCEVit and depression levels across four regression models with increasing covariate adjustment. Each arrow displays the corresponding Pseudo *R*^2^ and *p*-value, showing improved model fit with progressive adjustment.

The conceptual diagram of this regression model is represented in Figure 1.

These findings suggest that teachers in an “Excellent” ethical climate are less likely to experience depression. The robust correlation of a positive ethical climate supports the importance of fostering positive ethical standards in educational settings to improve teachers’ mental health. Hypothesis H2a, which is that ethical climate scores correlate with the depression dimension of the DASS-21 scale, was therefore accepted.

Since no significant correlation was found with anxiety hypothesis H2b was rejected.

Table 6 presents multinomial regression results examining the relationship between ethical climate (sCEVit scores) and stress levels (DASS-21) (hypothesis H2c). The model was used due to a significant univariate correlation, with “Good” as the baseline, as no participant scored “Poor.” Risk ratios (RR) and *p*-values for ethical climate categories are shown and adjusted for confounders such as gender, age, contract type, and classroom size. Results indicate a significant association between a “Discrete” ethical climate and increased stress levels. In the crude model (Model A), participants in the “Discrete” ethical climate category were more likely to experience stress (RR = 3.80, *p* = 0.014). This association remained significant across all adjusted models (Model B: gender and age; Model C: gender, age, and contract type; Model D: gender, age, contract type, and student number), suggesting the robustness of the relationship.

**Table 6:** Multinomial regression models for sCEVit scores and Stress subscale from DASS-21, pseudo *r*^2^ provides a global measure of model fit (significative correlation for crude and adjusted Stress scores to a “Discrete” or “Excellent” score at the sCEVit).

sCEVit	Pseudo *r*^2^	*p*-values	DASS-21 subscale	RRR	St. Err.	*t*-value	*p*-value	[95% Confidence interval]	Sig
Regression model									
Discrete	0.026	0.003	Stress^a^	3.798	2.055	2.47	0.014	1.315–10.969	**
	0.046	0.002	Stress^b^	3.273	1.813	2.14	0.032	1.106–9.692	**
	0.057	0.001	Stress^c^	3.317	1.846	2.15	0.031	1.115–9.874	**
	0.076	0.000	Stress^d^	3.435	1.943	2.18	0.029	1.134–10.409	**
Good (base level)									
Excellent			Stress^a^	0.616	0.182	−1.64	0.101	0.346–1.1	
			Stress^b^	0.57	0.173	−1.86	0.063	0.314–1.032	*
			Stress^c^	0.539	0.166	−2.01	0.045	0.295–0.986	**
			Stress^d^	0.487	0.153	−2.28	0.022	0.263–0.903	**

***p < 0.01, **p < 0.05, *p < 0.1.

a Crude.

b Adjusted for sex and age.

c Adjusted for sex, age, contract.

d Adjusted for sex, age, contract, student number.

For an “Excellent” ethical climate, the crude model did not reach significance (RR = 0.62, *p* = 0.101). However, with adjustments, a correlation emerged: Model B approached significance (RR = 0.57, *p* = 0.063), Model C reached significance (RR = 0.54, *p* = 0.045), and the fully adjusted Model D confirmed a negative correlation (RR = 0.49, *p* = 0.022).

**Figure 2:**
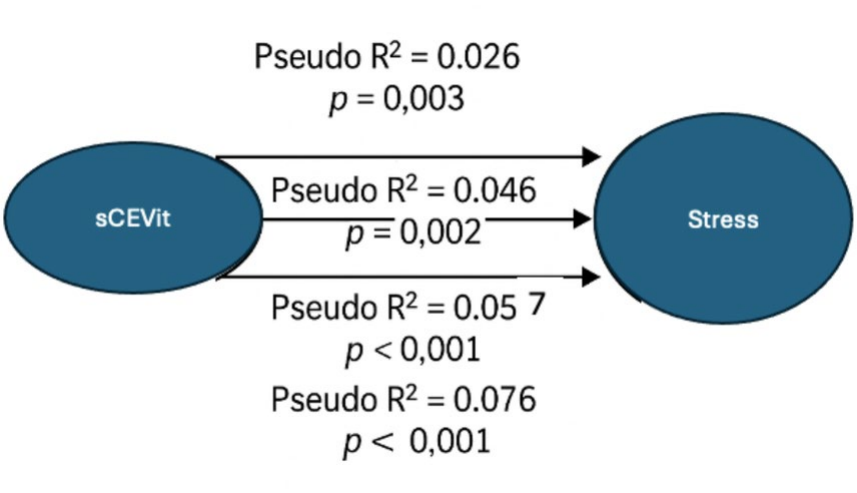
Conceptual diagram of the multinomial logistic regression model for Stress. The figure illustrates the association between sCEVit and stress levels across four regression models with increasing covariate adjustment. Each arrow displays the corresponding Pseudo *R*^2^ and *p*-value.

The conceptual diagram of this regression model is represented in Figure 2.

These findings suggest that a “Discrete” ethical climate is linked to higher stress, while an “Excellent” ethical climate significantly correlates to lower stress levels. This highlights the value of a positive ethical environment in supporting educators’ mental wellbeing. Hypothesis H2c, that ethical climate scores correlate with the stress dimension of the DASS-21 scale, was therefore accepted.

## 4 Discussion

This study investigates the relationship between workplace ethical climate (WEC) and teachers’ mental and physical health. Previous studies have demonstrated the effects of WEC on the quality of teaching, the relationship with students, and the overall wellbeing of the educational system (47). Despite these findings, little has been addressed concerning the teachers’ physical and mental wellbeing.

Findings from this study partially support Hypothesis 1, revealing that higher WEC levels (“Good” and “Excellent”) are negatively correlated with teachers’ stress and depression. However, no significant correlation was observed between WEC and anxiety. Contrary to Hypothesis 1b, no significant relationships were found between WEC and general health measures or sleep difficulties, emphasizing the nuances stemming from their perception of WEC. The current study aims to contribute to the existing literature on teachers’ health by focusing on their ethical climate perception. These surprising results could be explained by considering that non-linear relationships may link the studied variables. These results are consistent with previous scientific literature highlighting that poor ethical climate correlates with stress and depression in Italian workers (8). However, to the authors’ knowledge, this is the first study investigating this relationship in teachers. Consistent with results from this study, the relationship between poor ethical climate and worse mental health outcomes has been reported in many studies (10, 48), especially in healthcare workers (49–51), as highlighted by a recent systematic review (9). Furthermore, previous studies have highlighted how negative mental health outcomes associated with a poor ethical climate can reduce job satisfaction and lead to a higher turnover intention in workers (8, 10, 52). Evaluating these outcomes in teachers in order to assess if this association is significant could help improve their mental health as well as their overall wellbeing.

These connections to prior studies help situate the current research within the broader context of what we know about the relationship between organizational ethics and teacher wellbeing. It’s reassuring to see the patterns emerging across multiple investigations - a clear ethical framework and environment are important protective factors when preserving teachers’ mental health. This consistency lends credibility to the current study’s central conclusions. At the same time, identifying these links to past literature underscores this issue’s complexity and multifaceted nature. Many nuanced factors determine how a workplace’s ethical climate can impact individuals. Unpacking these dynamics more fully will be an important goal for future research. Recent studies support these findings, showing that teachers frequently face ethical dilemmas and perceive a lack of ethical support from their institutions, which correlated with higher stress levels and psychological distress (53).

Findings from this study support that a positive ethical climate, where teachers feel their values are respected and upheld, are negatively correlated to stress and depression. A mismatch between an employee’s values and the perceived values of the organization (in this case, the school and its teachers) can lead to cognitive dissonance, which is a source of stress and anxiety. Cognitive dissonance occurs when individuals experience discomfort due to conflicting beliefs and behaviors. Implementing company strategies or policies aimed at reducing the mismatch between company and personal values, could improve morale in teachers, leading to a better organizational climate (60).

The results partially confirmed Hypothesis 2, which confirmed the role of stress as a key factor correlated to teachers’ perceptions of the workplace ethical climate. Teachers working in ethically supportive environments reported a reduced risk of experiencing stress.

Results from this study did not highlight a significant correlation between ethical climate and anxiety in teachers. However, a statistically significant correlation between workplace ethics and anxiety has been highlighted in previous studies performed on white-collar workers (8). This may be due to a different effect of ethical climate or might be on teachers compared to other workers, or maybe due to the teachers included in the convenience sample for this study. Since the studies investigating the relationship between ethical climate and anxiety are very few, further research is necessary to define the effects of workplace ethics on teachers’ anxiety.

Similarly, the relationship between ethical climate and daytime sleepiness or general health has been scarcely investigated in previous scientific research, and emerged as not statistically significant in this study. This was surprising, as one might have assumed that a positive ethical environment would translate to better overall wellbeing. This unexpected result suggests that the influence of ethical climate may be more targeted, impacting specific mental health outcomes like depression and stress rather than having a broader impact on teachers’ physical and mental wellbeing. However, previous studies performed in other workers, have highlighted that workplace ethical climate can also be correlated to general health, physical health, and sleep disturbances (61), and a positive ethical climate has been correlated to better general health in white-collar workers (62). Despite this, scientific research in this area remains scarce, and most of the studies performed so far include health as a self-reported outcome (61). For these reasons, further research should focus on evaluating the relationship between ethical climate and overall health (general health, sleepiness, mental health, etc.) in teachers, focusing on objective measures of health obtained through medical examinations, and performing longitudinal studies, in order to clarify if a correlation does exist.

However, if the lack of a clear link between ethical climate and general health or sleep disturbances is confirmed by further research, it would highlight how complex factors contribute to a teacher’s overall health and wellness and that addressing the challenges educators face likely requires a multifaceted approach beyond simply focusing on the ethical culture of the workplace. This unexpected finding is a valuable lesson - it shows that the relationships between organizational factors and individual health are not always straightforward. It underscores the need to examine these issues from multiple angles and not make assumptions about how different elements may (or may not) be connected. Moving forward, a more nuanced and comprehensive perspective will be key to meaningfully supporting teachers’ wellbeing.

### 4.1 Limitations

This study has a few limitations. The main limitation of this study is its reliance on self-report tools, which means that the outcomes were assessed subjectively. Moreover, the cross-sectional design means that the self-reported, validated tools, were administered in a single session, which may have led to a common-method bias. Additionally, as participation was voluntary, there may be a selection bias if participants with poorer perceptions of workplace ethics felt less inclined to participate. However, this limitation was partially addressed by ensuring participant anonymity.

Furthermore, the cross-sectional design of this study means that establishing a causality relationship was not possible, although correlations analyses were significant. Future longitudinal study should contribute to this field by investigating the causality of these correlations.

The sample size was relatively small, and prevalently female, which could limit the generalizability of the results. Although the sample included various middle and high schools, participation in the study was on a voluntary basis, with a final sample of 255 teachers. Additional studies should be performed with larger samples, to assess the correlations highlighted in this study on a larger scale. Furthermore, the population was recruited through convenience sampling, which limits the generalizability of the results; therefore, a broader study encompassing multiple high schools may be necessary. This survey was carried out at a national level, therefore including only Italian schools, without international data. This may impact the generalizability of these results to a broader scale, such as the European or global context.

Another limitation of this study lies in the categorization of ethical climate scores into quartiles. While this approach enhances interpretability and practical applicability (particularly for practitioners, such as occupational safety officers and risk assessors) it introduces a degree of arbitrariness and may reduce statistical precision. Although continuous modeling would offer more robust statistical insight, the quartile structure was intentionally chosen to support the tool’s usability in real-world occupational settings. Future studies may benefit from complementary analyses using continuous scores.

### 4.2 Conclusion

This study highlights the crucial role of workplace ethical climate (WEC) in influencing teachers’ mental health, particularly regarding stress and depression. The findings show that a positive ethical climate is associated with lower mental health challenges, suggesting that enhancing the ethical standards in educational environments can significantly benefit teacher wellbeing.

Interestingly, no significant correlations were found between WEC and general health or sleep disturbances, suggesting that while ethical climate is a key factor in psychological wellbeing, it may not directly impact other dimensions of health. This distinction highlights the complexity of the factors influencing overall health, suggesting the need for a multidimensional approach to health promotion within educational settings.

Given the evidence linking ethical climate with reduced stress and depression, educational policymakers and school administrators should prioritize creating and maintaining supportive and ethically sound work environments. Such efforts can promote a healthier workplace, increase job satisfaction, and improve teacher retention, benefiting the entire educational system.

Future research should aim to build on these findings by employing longitudinal study designs and incorporating objective health measures to validate and expand the current insights. This will provide a more comprehensive understanding of how ethical climate interacts with various aspects of health and wellbeing, helping to inform more effective interventions and policies in the educational context. Additionally, exploring moderating variables such as organizational support, leadership styles, or school climate will provide a deeper understanding of the mechanisms underlying these relationships. These investigations can guide the development of targeted interventions to improve ethical climate and assess their impact on teacher outcomes.

## Data Availability

The raw data supporting the conclusions of this article will be made available by the authors, without undue reservation.
